# Supplementary material for: Plasma Interleukin-10 and Cholesterol Levels May Inform about Interdependences between Fitness and Fatness in Healthy Individuals
Source: Int J Environ Res Public Health. 2021 Feb 12;18(4):1800. doi: 10.3390/ijerph18041800 (PMC7917930; doi:10.3390/ijerph18041800)
Supplement: Supplementary file 1 [file ijerph-18-01800-s001.pdf]

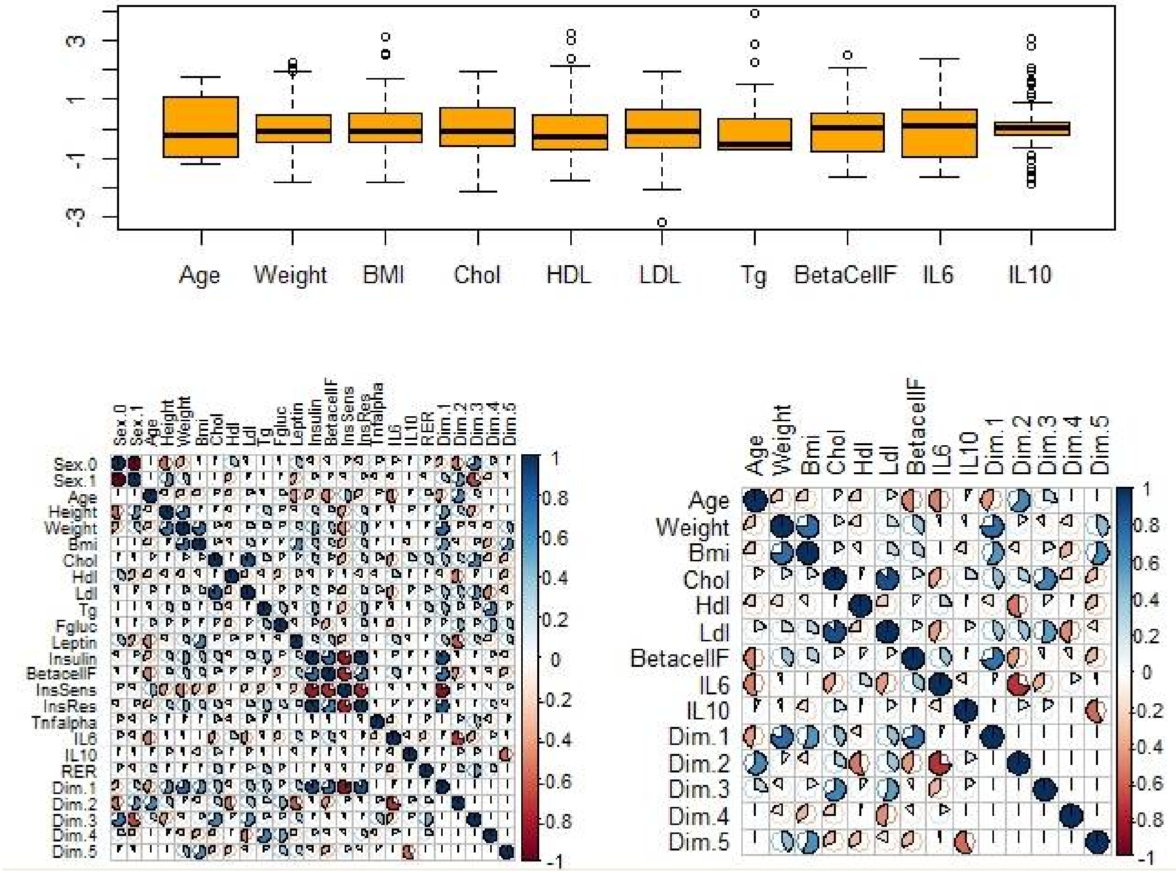

Figure S1. Boxplot of selected variables, and correlation matrices before and after the selection.
